# 

**Published:** 1888-09

**Authors:** 


					﻿NOTICE.
All articles for publication, books for review, business communications, etc.,
must'be sent to Editors, 1123 Spruce Street, Philadelphia.
Subscribers will please notice that this Journal will be sent until arrears
are paid and it is ordered discontinued.
In writing to your friends, be sure to advise them of the
work the HOMEOPATHIC PHYSICIAN is doing. The
more subscribers, the larger and the better journal!
i( Am I my brother’s keeper?” You are to the extent of
being negligent of his best interests if you let him be igno-
rant of the great work THE HOMCEOPATHIC PHYSI-
CIAN is doing. See that your friends know of this work.
				

